# Wine microbiology is driven by vineyard and winery anthropogenic factors

**DOI:** 10.1111/1751-7915.12428

**Published:** 2016-10-25

**Authors:** Cédric Grangeteau, Chloé Roullier‐Gall, Sandrine Rousseaux, Régis D. Gougeon, Philippe Schmitt‐Kopplin, Hervé Alexandre, Michèle Guilloux‐Benatier

**Affiliations:** ^1^ Univ. Bourgogne Franche‐ComtéAgroSup DijonPAM UMR A 02.102F‐21000DijonFrance; ^2^ IUVV Equipe VAlMiSrue Claude LadreyBP 2787721078Dijon CedexFrance; ^3^ Chair of Analytical Food ChemistryTechnische Universität MünchenAlte Akademie 1085354Freising‐WeihenstephanGermany; ^4^ Research Unit Analytical BioGeoChemistryDepartment of Environmental SciencesHelmholtz Zentrum MünchenIngolstaedter Landstrasse 185764NeuherbergGermany; ^5^ IUVV Equipe PAPCrue Claude LadreyBP 2787721078Dijon CedexFrance

## Abstract

The effects of different anthropic activities (vineyard: phytosanitary protection; winery: pressing and sulfiting) on the fungal populations of grape berries were studied. The global diversity of fungal populations (moulds and yeasts) was performed by pyrosequencing. The anthropic activities studied modified fungal diversity. Thus, a decrease in biodiversity was measured for three successive vintages for the grapes of the plot cultivated with Organic protection compared to plots treated with Conventional and Ecophyto protections. The fungal populations were then considerably modified by the pressing‐clarification step. The addition of sulfur dioxide also modified population dynamics and favoured the domination of the species *Saccharomyces cerevisiae* during fermentation. The non‐targeted chemical analysis of musts and wines by FT‐ICR‐MS showed that the wines could be discriminated at the end of alcoholic fermentation as a function of adding SO_2_ or not, but also and above all as a function of phytosanitary protection, regardless of whether these fermentations took place in the presence of SO_2_ or not. Thus, the existence of signatures in wines of chemical diversity and microbiology linked to vineyard protection has been highlighted.

## Introduction

For over 7500 years, humans have sought to control vine development, grape berry maturation and alcoholic fermentation to produce wine (McGovern *et al*., 1996). Over the last 20 years, the emergence of various vineyard management methods has been observed, particularly with the increasing number of vineyards practising organic viticulture (Zafrilla *et al*., 2003). This diversity and especially the use of chemical or organic phytosanitary products could affect the biodiversity of grape microorganisms. Various studies have been conducted to compare the effects of these different systems and more particularly the non‐target effects of phytosanitary treatments on fungal populations present on berries. Although all these studies show an effect of plant protection on the diversity of yeasts present in grape berries, the results cannot be generalized and are very often contradictory. Cordero‐Bueso *et al*. (2011) and Martins *et al*. (2014) observed a wider diversity of yeasts for organic plots compared with conventional plots. Regarding the study by Milanović *et al*. (2013), the lowest diversity of isolated yeasts was observed for the organic modality. At genus or species level, Guerra *et al*. (1999) observed that the species *Saccharomyces cerevisiae* was not isolated in the conventional modality compared with the organic modality. The fermentative yeast genera such as *Hanseniaspora* and *Metschnikowia* have been isolated mainly in control (untreated) and organic plots, whereas *Aureobasidium pullulans* was the majority species isolated in grape berries from conventional plots (Comitini and Ciani, 2008). But in another study, the species *A. pullulans* was described as the majority species isolated in grape berries from organic plots (Martins *et al*., 2014) and the species *Metschnikowia pulcherrima* was isolated more in samples obtained from the conventional modality (Milanović *et al*., 2013). Significant variability between studies is likely due to differences in grape varieties, the geographical location of the vineyard, the sampling method, identification techniques and finally intra‐vine variation (plot level) (Hierro *et al*., 2006; Xufre *et al*., 2006; Nisiotou *et al*., 2007; Barata *et al*., 2008, 2012; Setati *et al*., 2012, 2015). Furthermore, *in vitro* studies are still required to determine the sensitivity of different fungal genera to these products as well as studies in the vineyard to determine the direct effect of the products used at the time of application. For Cadez *et al*. (2010), the presence of fungicides has a minor impact on yeast communities associated with grape berries because, after the safety interval, colonization with yeast is possible.

In addition, such works do not consider whether the differences of yeast biodiversity observed as a function of plant protection are maintained in the musts and during alcoholic fermentation. Indeed the pre‐fermentation operations carried out to ensure the quality of the final product could reduce or on the contrary amplify the differences observed for yeast biodiversity in the vineyard. For musts obtained from white grapes (Chenin Blanc and Prensal White), cold settling reduces the overall yeast population and particularly affects the growth of certain species such as *Hansenula anomala*,* Issatchenkia terricola* and *S. cerevisiae*, instead of other species such as *Candida zemplinina* and *Hanseniaspora uvarum*, not very sensitive to this process and which become or remain the major species after racking (Mora and Mulet, 1991). Sturm *et al*. (2006) observed for Riesling grape must that non‐*Saccharomyc*es yeasts persist longer during fermentation if pressing is preceded by crushing or maceration. The temperature during pre‐fermentation maceration of red grape varieties (Cabernet sauvignon and Malbec) also seems to play a role in the evolution of yeast populations (Maturano *et al*., 2015). Thus, maceration carried out at 14°C resulted in the development of yeast populations with a high proportion of *H. uvarum*. For maceration performed at 2.5 or 8°C, yeast populations did not increase but the proportions of *S. cerevisiae* and *C. zemplinina* increased at 8 and 2.5°C respectively. These results show the strong preference of the species *H. uvarum* for temperatures around 15°C and confirm the psychrotolerant characteristic of the species *C. zemplinina* (Sipiczki, 2003). The addition of SO_2_ in red and white wine promotes the establishment of *S. cerevisiae*, often to the detriment of non‐*Saccharomyces* yeasts (*Candida*,* Cryptococcus*,* Hanseniaspora* and *Zygosaccharomyces*) more sensitive to SO_2_, and also less tolerant to ethanol (Romano and Suzzi, 1993; Constantí *et al*., 1998; Henick‐Kling *et al*., 1998; Albertin *et al*., 2014; Takahashi *et al*., 2014). Bokulich *et al*. (2015) studied the effect of different concentrations of SO_2_ (between 0 and 150 mg l^−1^) on bacterial and fungal populations during the alcoholic fermentation of Chardonnay grape musts. Fermentations were slower and extended with low SO_2_ concentrations (< 25 mg l^−1^) due to the growth of bacteria or fungi competing with yeasts, but the development of bacterial and fungal species was greatly reduced with the addition of 25 mg l^−1^ SO_2_. However, higher concentrations up to 100 mg l^−1^ had no additional effect on populations or on the progress of fermentation. Beyond this concentration, fermentation was slower than those conducted with concentrations between 25 and 100 mg l^−1^. The effects of four pre‐fermentation oenological practices (clarification degree to 90 NTU and 250 NTU), temperature during pre‐fermentation maceration (10 and 15°C), the use of SO_2_ (0 and 25 mg l^−1^) and starter yeast addition on yeast dynamics (*C. zemplinina*,* Hanseniaspora* spp., *Saccharomyces* spp. and *Torulaspora delbrueckii*) were evaluated in a Chardonnay grape must (Albertin *et al*., 2014). The population dynamics of the four species were impacted differently by oenological practices. For example, the use of SO_2_ seemed to favour the genus *Saccharomyces* independently of other practices. Significant interaction effects between practices were revealed. Thus, a low degree of clarification seemed to favour the development of *C. zemplinina* mainly at a pre‐fermentation temperature of 10°C. The inhibition of the genus *Hanseniaspora* was observed at a pre‐fermentation temperature of 10°C in the presence of SO_2_.

Although the dynamics of yeast populations in the composition and organoleptic qualities of wine is an important parameter (Lambrechts and Pretorius, 2000; Swiegers *et al*., 2005), must composition is also a parameter that cannot be neglected. Does the composition of musts and wines differ according to the phytosanitary protection used? To our knowledge, few studies have been performed on this subject. Existing studies have focused their analyses on compounds related to natural plant defences as they are considered as the compounds directly affected by plant protection (Adrian *et al*., 1997; Jeandet *et al*., 2000). Thus, the concentrations of polyphenols and antioxidant activity were higher for berries sampled from an organic plot 30 days before harvesting. However, these differences disappeared at harvest (Mulero *et al*., 2010). On the contrary, Bunea *et al*. (2012) found a small difference in total polyphenol contents between berry skins for nine different grape varieties from conventional and organic vineyards (respectively 148–1231 and 163–1341 mg kg^−1^ as gallic acid equivalents). Dani *et al*. (2007) also showed that the concentration of total polyphenols, particularly resveratrol, is higher for musts elaborated with grape varieties from *Vitis labrusca* (Bordo and Niagara) from organic viticulture. Levite *et al*. (2000) compared the concentration of resveratrol for conventional and organic wines made from five grape varieties and from six geographical localizations. In most cases, the resveratrol concentration was higher for organic wines. Similarly, Vrček *et al*. (2011) observed a higher concentration of polyphenols for wines from organic viticulture compared with those from conventional viticulture.

The effects of plant protection applied to vineyards on wine composition have been studied mainly from a sensory point of view and are not clearly defined (Moyano *et al*., 2009; Pagliarini *et al*., 2013). In addition, most of these studies have not taken into account practices used during winemaking, which can reduce or increase differences due to the protection applied to the vineyard. Finally, to our knowledge no study has determined how the modifications of microbial populations due to plant protection may influence wine composition.

The purpose of this work was to study the combined impacts of three different phytosanitary protections: Organic, Conventional and Ecophyto protections (corresponding to dose reduction compared with conventional treatment); and of oenological practices (pressing‐settling and sulfiting) on the biodiversity of fungal populations and on the chemical composition of musts and wines. For the first time, the effects of phytosanitary protection on microbiological and chemical characteristics were evaluated from grape berries to wine in a single study. The use of non‐target methods for microbiological (pyrosequencing) and metabolomic (FT‐ICR‐MS) analyses allowed examining the global effects of three different phytosanitary protections. However, only the non‐volatile fraction of the wine composition has been considered in our approach.

## Results and discussion

### Effect of the phytosanitary protection on fungal populations present on grape berries for the 2012, 2013 and 2014 vintages

**Figure 1:**
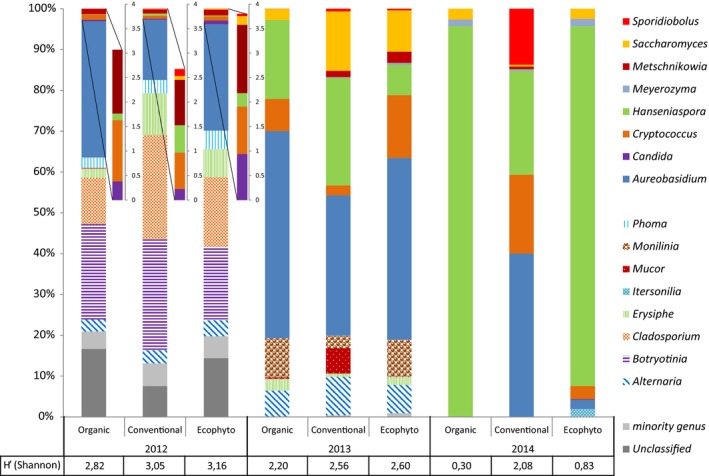
Repartition on fungal genera on grape berries (T0) for vineyard with Organic, Conventional and Ecophyto phytosanitary protection. Populations are identified by pyrosequencing for 2012, 2013 and 2014 vintage. H′ index are calculated on overall population. Genera representing < 0.2% of the total population are collectively called ‘minority genera’.

The fungal populations present on mature Chardonnay berries (after pressing aseptically) and identified by pyrosequencing for the three blocks treated with different phytosanitary protections and for the three vintages are presented in Fig. 1. Moulds and yeasts were identified by pyrosequencing. As observed by Nisiotou *et al*. (2007) with a culture‐dependent method for identifying fungal populations, the highest fungal diversity was observed when moulds were present in quantity. This was also confirmed by the Shannon biodiversity index which was higher in 2012 compared with the other two vintages, regardless of the protection applied. The proportion of moulds seemed primarily related to vintage: between 42% and 70% of the population for the 2012 vintage, between 18% and 19% for the 2013 vintage and < 2% for the 2014 vintage. This difference in the proportion of mould according to vintage is probably related to differences in temperature and rainfall between vintages during the flowering‐harvest period (Sall, 1980; Lalancette *et al*., 1988; Broome *et al*., 1995). Indeed for the 2012 vintage, the average temperature for this period was 20.1°C with a rainfall of 306 mm, whereas the average temperature was 19.4°C in 2013 and 2014, with rainfalls of 271 and 244 mm for 2013 and 2014 respectively. Some mould genera appeared to be specific to one vintage. For example, *Botryotinia, Cladosporium* and *Phoma* genera were present only for the 2012 vintage (for the three different protections), while the genus *Monilinia* (for the three types of protection) was present only for the 2013 vintage. Moreover, the number of yeast genera identified was much higher for the 2012 vintage compared with the other two modalities which had a lower proportion of mould. Some yeast genera seem to be linked to the presence of certain moulds. For example, the genus *Candida,* known to be very present on botrytized grapes (Mills *et al*., 2002; Sipiczki, 2003), was only identified in our study for the 2012 vintage, the only vintage in which the genus *Botrytis* was present. Moreover, the proportions of the genus *Hanseniaspora*, known for its antagonism with *Botrytis cinerea* (Rabosto *et al*., 2006; Liu *et al*., 2010), and of the genus *Saccharomyces*, sensitive to glucan produced by *B. cinerea* (Hidalgo, 1978; Donèche, 1993), were much lower in 2012 compared with the other two vintages. It is interesting to note that the genus *Saccharomyces* (2012 vintage) may have been present in amounts too small to be detected by standard methods (0.2% and lower than 0.1% for the Ecophyto and Conventional modalities respectively) or even completely absent (0% in the Organic modality). It has long been known that this genus is difficult to isolate from grapes (Combina *et al*., 2005; Raspor *et al*., 2006). These three yeast genera, *Saccharomyces, Hanseniaspora and Candida,* have been described as having a strong influence on the quality and organoleptic profile of wines (Zironi *et al*., 1993; Ciani and Maccarelli, 1998; Andorrà *et al*., 2010; Moreira *et al*., 2011). Thus, the modulation of the yeast populations on grape berries by the presence of some phytopathogenic genera could have effects on wine products.

The Shannon index highlighted a plant protection effect: the Organic modality had a lower biodiversity index compared with the other two modalities regardless of the vintage. For the 2014 vintage, the Shannon index was 0.30 for the Organic modality and 0.80 and 2.08 for the Ecophyto and Conventional modalities respectively. Milanović *et al*. (2013) also observed that Organic protection could lead to lower biodiversity on berries compared with Conventional protection. However, these authors studied only species of yeasts. The results obtained in this study suggest that interactions could exist and that the presence of certain fungal genera may promote or inhibit the presence of certain yeast genera. However, this is not enough to explain the lowest biodiversity systematically observed for the Organic modality. The latter could be related to an effect of copper on non‐target organisms (e.g. yeast). Copper is a fungicide with a broader spectrum than the synthetic molecules used in the other two protection modalities. Thus, Martins *et al*. (2014) have recently observed a strong correlation between the copper dose used and the decrease of yeast biodiversity observed on berries. In this study, the amounts of copper applied (kg per ha) are: 2.67 for vintage 2012 and 2013, 1.89 for vintage 2014 in the Organic modality, 0.5 for vintage 2012 and 2013, 0.39 for vintage 2013 in the Conventional modality, and 0.25 for vintage 2012, 0 for vintage 2013 and 0.1 for vintage 2014 in the Ecophyto modality.

Furthermore, it is interesting to note that the genus *Sporidiobolus,* representing 13.7% of the population on berries for the Conventional modality, was not detected for the other two modalities. This can be explained partly by the high resistance of this genus to synthetic fungicides (Sláviková and Vadkertiová, 2003) and its sensitivity to copper (Vadkertiová and Sláviková, 2006).

The fungal populations present on berries appeared to result from both the protection and the vintage. The amount of phytosanitary products varied each year (Table S1) regardless of the type of protection. This quantity depended on disease pressure and the risk of leaching related to annual climatic conditions. This difference in disease pressure was very marked between the three vintages studied and caused significant effects on the overall fungal populations present on the berries, and not only on the proportion of the different plant pathogens. Nevertheless, more than the dose used, it was the type of molecules used that seemed to have a very significant effect on the diversity of fungal genera present in berries. Thus, the grape berries of the Organic modality using copper and sulfur fungicides always presented the lowest biodiversity compared with the other two types of protection using synthetic molecules.

### Impact of pre‐fermentation steps and vineyard protection on grape musts for the 2013 vintage

#### Fungal populations

Fungal populations present in the must after pressing‐settling (T1) were compared with those present on berries (T0). The results are shown in Table 1. The population of moulds decreased significantly in the grape must after the pressing‐settling step. Thus, the genus *Alternaria,* which represented 6.4%, 7% and 9.4% of the total fungi population on the berries of the Organic, Ecophyto and Conventional modalities, respectively, was not found in the musts. The same decrease was observed for the genera *Monilinia* and *Erysiphe*. On the contrary, the genus *Penicillium*, not identified on berries, was represented in must as 2.36% of the total fungi population for the Ecophyto modality and 0.08% for the Organic modality, probably due to the implantation of strains present in the cellar environment. Ocón *et al*. (2011) had already highlighted that this genus was mostly detected in the cellar environment. The Conventional modality differed from the other two modalities by the total absence of mould.

**Table 1:** Repartition (%) of fungal genera identified by pyrosequencing on berries (T0) and after pressing‐settling (T1) for three phytosanitary vineyard protections for 2013 vintage

	O		C		E	
	T0	T1	T0	T1	T0	T1
Mould						
*Alternaria*	6.37	–	9.45	–	7.01	–
*Erysiphe*	2.90	0.02	0.85	–	1.91	0.04
*Fusarium*	0.10	0.03	0.24	–	0.74	–
*Itersonilia*	0.04	0.02	0.02	–	–	–
*Monilinia*	9.56	0.05	3.00	–	9.11	0.68
*Mucor*	0.27	0.06	6.15	–	–	–
*Penicillium*	–	0.08	–	–	–	2.36
Yeast						
*Aureobasidium*	50.77	18.59	34.39	5.59	44.56	54.91
*Candida*	–	0.10	–	0.32	–	0.02
*Cryptococcus*	7.86	48.10	2.47	11.19	15.37	20.89
*Debaryomyces*	–	0.03	–	0.11	–	0.02
*Hanseniaspora*	19.39	17.96	26.36	27.02	7.46	2.73
*Kazachstania*	–	0.03	–	0.08	–	0.04
*Malassezia*	–	0.02	–	–	–	–
*Metschnikowia*	–	0.02	1.54	24.97	2.69	3.49
*Meyerozyma*	–	5.26	0.17	5.03	0.49	1.08
*Saccharomyces*	2.71	9.64	14.57	25.71	10.17	6.70
*Sporidiobolus*	0.02	–	0.63	–	0.39	6.54
Unclassified	–	–	0.15	–	0.12	0.47

The number of yeasts (CFU ml ^−1^) was lower in the three modalities in T0 samples than for T1 samples (Table 2). This difference could be related to sampling and pressing methods: the quantities of berries were lower at T0 compared with T1; berries at T0 were pressed manually and those of the harvest at T1 were pressed with a vertical press.

**Table 2:** Yeast count on YPD medium for berries (T0) and grape must after pressing and after settling for three phytosanitary vineyard protections for 2013 vintage. Values followed by different letters are significantly different (*P* < 0.01)

	O			C			E		
	T0	After pressing	After settling	T0	After pressing	After settling	T0	After pressing	After settling
Yeast log CFU ml^−1^ (standard deviation)	4.01 (2.48)	5.49^a^ (3.92)	4.93^b^ (3.68)	3.59^c^ (2.42)	5.27^a^ (3.48)	5.17^b^ (4.33)	3.82^c^ (2.18)	5.59^a^ (3.30)	5.10^b^ (4.06)

The decrease of yeast populations was also observed during the settling step for all the musts whatever the type of protection applied to the vineyard (Table 2). This reduction of yeast populations confirmed the results of Mora and Mulet (1991), who reported a decrease in cell number for several yeast genera during this step. Not only a decrease of total yeast populations was observed after pressing‐settling compared to the proportion on berries but a difference was also observed in the proportion mainly for *Aureobasidium* genera. Moreover, some genera present in musts (*Candida, Debaryomyces, Kazachstania* and *Malassezia*) were not present on berries. This could be due to the implantation of the resident flora from the cellar during the pressing‐settling steps (Grangeteau *et al*., 2015). Nevertheless, the genus *Candida* represented < 0.3% of the population, while the genera *Debaryomyces, Kazachstania* and *Malassezia* represented < 0.1%. The implantation of resident flora from the cellar in the must could also explain the increase in the proportions of the genera *Cryptococcus, Metschnikowia, Meyerozyma* and *Saccharomyces* between berries and grape musts. Otherwise, some genera were affected differently by this step as a function of the protection modality applied in the vineyard. For example, the genus *Aureobasidium* which represented 50.8%, 34.4% and 44.6% of the population on berries for the Organic, Conventional and Ecophyto modalities, respectively, was found in musts at 18.6%, 5.66% and 54.9% for the Organic, Conventional and Ecophyto modalities respectively (Table 1).

**Figure 2:**
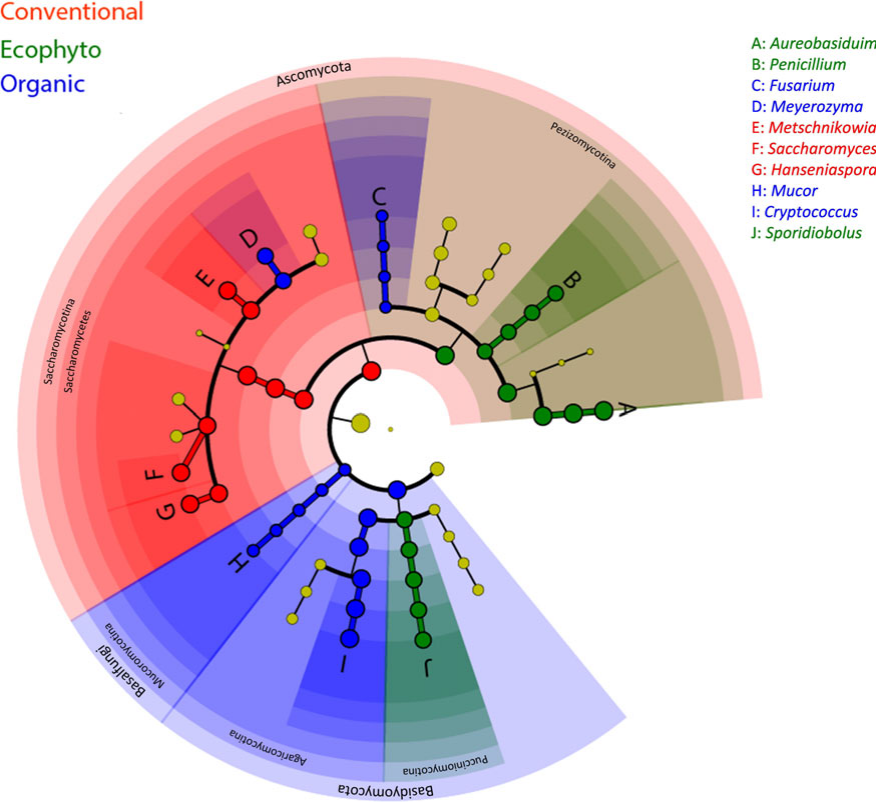
Least discriminant analysis effect size taxonomic cladogram comparing all grape musts categorized by vineyard protection mode. Significantly discriminant taxon nodes are coloured and branch areas are shaded according to the highest‐ranked variety for that taxon. For each taxon detected, the corresponding node in the taxonomic cladogram is coloured according to the highest‐ranked group for that taxon. If the taxon is not significantly differentially represented between sample groups, the corresponding node is coloured yellow.

A least discriminant analysis effect size (LDA) taxonomic cladogram comparing all the grape musts categorized by the different vineyard protections was performed (Fig. 2) to determine whether, despite these population reshuffles, a specific population was present depending on plant protection. Basidiomycota [especially *Cryptococcus* (48.1%)] were mainly associated with the Organic protection, while Ascomycota, including *Saccharomycotina* [especially *Saccharomyces* (25.7%), *Metschnikowia* (25%) and *Hanseniaspora* (27%)] were mainly associated with the Conventional protection. Among the Ascomycota, *Pezizomycotina* [especially *Aureobasidium* (54.9%)] were associated with the Ecophyto protection. *Fusarium* and *Mucor* (only 0.03% and 0.06% respectively) were associated with the Organic protection because they were not detected in the other modalities. *Penicillium* was associated with the Ecophyto protection even though the source of its presence was probably the cellar. Note that some differences observed for grape must can be related to those observed on grape berries. Indeed, *Hanseniaspora* and *Saccharomyces* represented a larger proportion of the population on berries for the Conventional modality (26.4% for *Hanseniaspora* and 14.6% for *Saccharomyces*) compared with the other modalities (*Hanseniaspora* 19.4% and 7.5% for the Organic and Ecophyto modalities, respectively, and *Saccharomyces* 2.7% and 10.2% for the Organic and Ecophyto modalities respectively). The genera *Cryptococcus* and *Aureobasidium* represented a lower proportion of the population on berries for the Conventional modality (34.4% for *Aureobasidium* and 2.5% for *Cryptococcus*) compared with the other modalities (*Aureobasidium* represented 50.8% and 44.6% for the Organic and Ecophyto modalities, respectively, and *Cryptococcus* represented 7.9% and 15.4% for the Organic and Ecophyto modalities respectively).

#### Chemical composition

**Figure 3:**
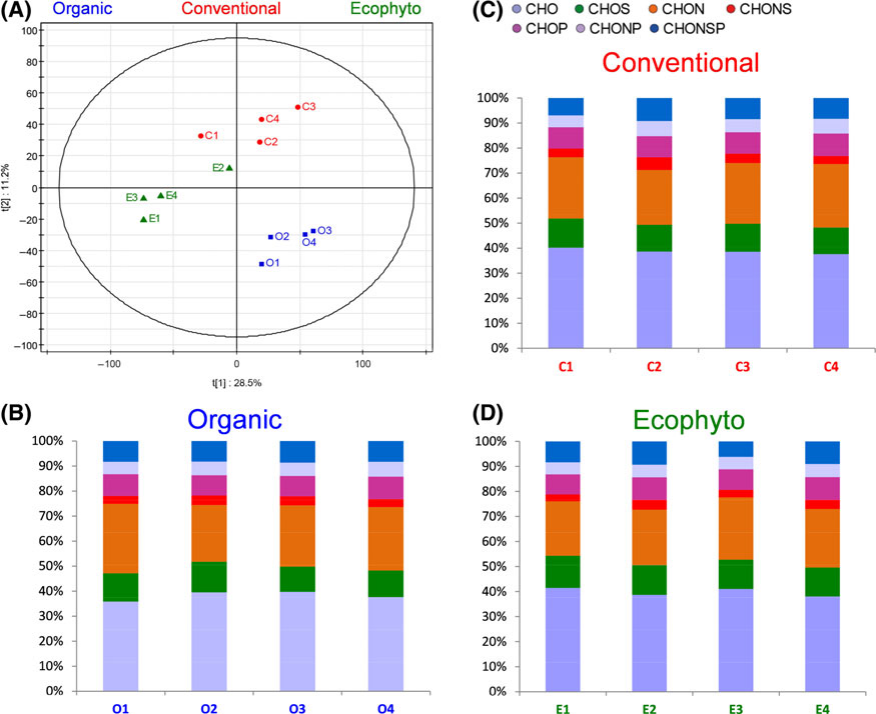
Analysis of the FT‐ICR–MS data for grape musts of 2013 vintage. (A) Scores plot of the PLS‐DA depending on the phytosanitary protection mode, the first two components retained 39.7% of the variation. Histograms of elementary composition of Organic (B), (C) Conventional and (D) Ecophyto grape musts.

The chemical compositions of the three different musts from the 2013 vintage were analysed (Table S3). No significant difference between the musts concerning sugar concentration or acidity level (pH or total acidity) was observed. The available nitrogenous compound content differed slightly depending on the must but was higher than 140 mg N l ^−1^ for all them, so there was *a priori* no deficiency preventing the progress of alcoholic fermentation (Agenbach, 1977). Taking the study still further, the grape musts were analysed by FT‐ICR‐MS. Distributions of CHONSP containing elemental compositions were extremely close for all the musts (Fig. 3B–D), explained by the fact that the grape berries of each must had the same origin. From these results, we can conclude that the direct influence of plant protection on the composition of the musts was quite low. Nevertheless, PLS‐DA (Fig. 3A) allowed partial discrimination of the musts as a function of protection. The grape musts of the Organic modality were separated from the musts of the other two modalities. Associated with the differences between the populations already shown, they could lead to differences in the dynamics of the alcoholic fermentation and chemical composition of wines depending on the phytosanitary protection applied in the vineyard.

### Impact of sulfur dioxide use and plant protection on wine during alcoholic fermentation for the 2013 vintage

#### Fungal populations

**Figure 4:**
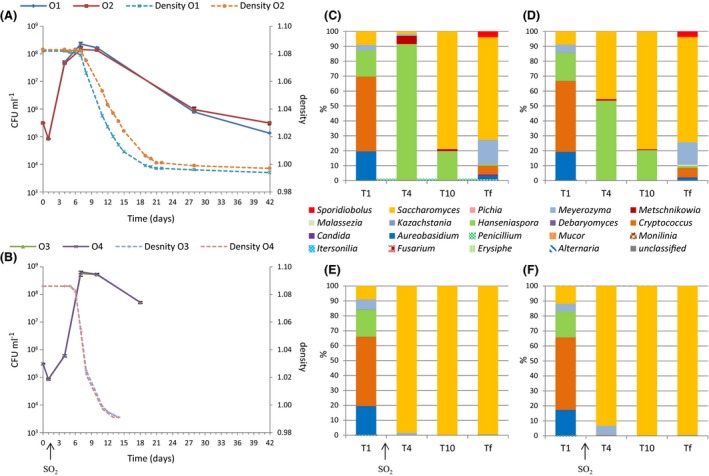
Monitoring of grape must fermentation of Organic modality for 2013 vintage. Monitoring of yeast population size (CFU ml^−1^) and fermentation progress (density) without SO2 (A) and with SO2 (B). Repartition of fungal genera during alcoholic fermentations without SO2 for must O1 (C) and must O2 (D) and with SO2 for must O3 (E) and must O4 (F). Populations are identified by pyrosequencing 1 day (T1), 4 days (T4), 10 days (T10) and at end of AF.

**Figure 5:**
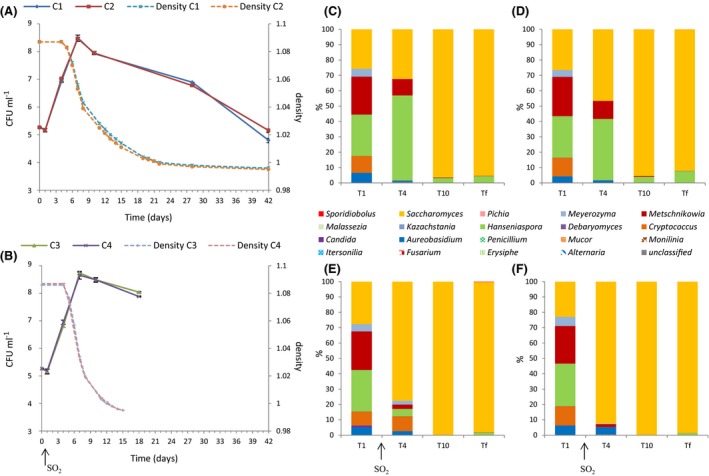
Monitoring of grape must fermentation of Conventional modality for 2013 vintage. Monitoring of yeast population size (CFU ml^−1^) and fermentation progress (density) without SO2 (A) and with SO2 (B). Repartition of fungal genera during alcoholic fermentations without SO2 for must C1 (C) and must C2 (D) and with SO2 for C3 (E) and C4 (F) musts. Populations are identified by pyrosequencing at 1 day (T1), 4 days (T4), 10 days (T10) and at the end of AF (Tf).

**Figure 6:**
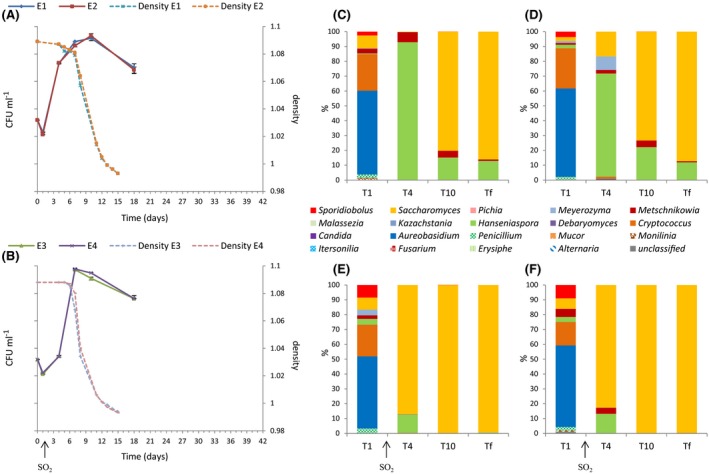
Monitoring of grape must fermentation of Ecophyto modality for 2013 vintage. Monitoring of yeast population size (CFU ml^−1^) and fermentation progress (density) without SO2 (A) and with SO2 (B). Repartition of fungal genera during alcoholic fermentations without SO2 for must E1 (C) and must E2 (D) and with SO2 for must E3 (E) and must E4 (F). Populations are identified by pyrosequencing 1 day (T1), 4 days (T4), 10 days (T10) and at end of AF (Tf).

The evolution of the number and proportion of yeast populations during AF are presented for the Organic, Conventional and Ecophyto modalities in Figs 4, 5, 6 respectively. For the Organic and Conventional modalities, fermentations in the absence of SO_2_ languished. The maximal population was reached after 7 days for the Conventional modality, with the same kinetics in the presence and absence of SO_2_. For the Organic modality, the maximal population was also reached after 7 days, but the kinetics and the maximum population level differed as a function of the presence or absence of sulfites. Indeed, in the absence of SO_2_, the population increased more quickly but the maximum population was statistically lower: 3. 10^8^ CFU ml^−1^ without SO_2_ versus 8.10^8^ CFU ml^−1^ with SO_2_ (Student's test with *P*‐value = 0.0007). These differences in behaviour may be related to the difference in composition of the yeast population present in the must. For the Ecophyto modality, no difference was observed concerning fermentation in the absence of SO_2_. The end of alcoholic fermentations occurred simultaneously for the four wines from this modality.

The proportion of *Saccharomyces* increased rapidly in the presence of SO_2_ and for the three modalities. It accounted for 80–98% of the population after 3 days of alcoholic fermentation. Thus, the differences in the population dynamics were limited in the presence of SO_2_. However differences in the initial population, even in the cases where they disappear quickly, can influence the finished wine (Romano *et al*., 2003). The genus *Saccharomyces* prevailed in all the wines at the end of AF. Without SO_2_, the implantation of *Saccharomyces* was delayed, so differences of populations could persist longer. Additionally, the population of non‐*Saccharomyces* remained high at the end of AF for the Organic (30%) and Ecophyto (15%) modalities. For the Conventional modality, *Saccharomyces* represented more than 95% of the total population after 9 days. Alcoholic fermentation was sluggish for the Organic modality. This sluggish fermentation could be due to dead or inactive populations or an external contamination because the *Aureobasidium*,* Cryptococcus*,* Meyerozyma* and *Sporidiobolus* genera were not usually identified at the end of AF. Differences in the *Saccharomyces* strains present during AF could explain differences in alcoholic fermentation dynamics. Furthermore, population size is an important factor for AF dynamics (Albertin *et al*., 2011). For the Organic and Ecophyto fermentations, a higher percentage and longer persistence of the genus *Hanseniaspora* could explain the difficulties of the development of the genus *Saccharomyces* (Medina *et al*., 2012). This genus never represented more than 85% of the total population in Organic and Ecophyto modalities. Otherwise, the presence and persistence of the *Hanseniaspora* and *Metschnikowia* genera for fermentations without SO_2_ could strongly influence the chemical composition of wines produced. Indeed species belonging to the *Hanseniaspora* genus and the *Metschnikowia* genus are known to produce secondary metabolites which can influence the organoleptic profiles of the wines produced negatively (Ciani and Picciotti, 1995) or positively (Zironi *et al*., 1993; Rojas, 2003; Moreira *et al*., 2011; Sadoudi *et al*., 2012; Medina *et al*., 2013; Martin *et al*., 2016).

#### Chemical composition

The question is whether these differences in population dynamics (in both number and composition) had an impact on the chemical composition of wine products and whether the differences observed in grape must between the Organic modality and the two other modalities persisted. Wine composition is reported in Table 3. The higher concentrations of ethanol found in the Ecophyto modality can be explained by the complete fermentation observed for the four batches. For the Organic modality, the difference between batches with and without SO_2_ can also be explained by the presence or absence of residual sugars. However, differences can be noted between the wines of the three modalities and so depending on the protection of the vineyard. The sugar concentrations of all the musts were very close, leading to the assumption that the presence of fermenting sugars at the end of AF was linked to the lower fermentative capacity of some populations related to differences between the strains of *S. cerevisiae* present or to the presence of non‐*Saccharomyces* yeast, particularly for wine fermented without SO_2_ (Charoenchai *et al*., 1998; Bisson, 1999; Zohre and Erten, 2002; Ferreira *et al*., 2006).

**Table 3:** Analytical characteristics of the wines elaborated from grape berries harvested in three phytosanitary vineyard protections and fermented with or without SO_2_ for 2013 vintage

	Wines	Alcoholic degree (% v/v)	Residual sugars (g l^−1^)	l‐malic acid (g l^−1^)	Volatile acidity (g acetic acid l^−1^)	Total SO_2_ (mg l^−1^)
−SO_2_	O1	12.55	2.1	2.5	0.68	4
	O2	12.45	2.0	2.3	0.68	5
+SO_2_	O3	12.80	< 1	2.6	0.50	10
	O4	12.75	< 1	2.6	0.52	10
−SO_2_	C1	12.80	3.2	2.2	0.78	3
	C2	12.70	4.0	2.3	0.77	5
+SO_2_	C3	12.90	3.0	1.9	0.42	9
	C4	12.90	3.3	2.6	0.42	11
−SO_2_	E1	13.00	< 1	2.1	0.36	5
	E2	13.1	< 1	2.1	0.34	3
+SO_2_	E3	13.15	< 1	2.1	0.22	10
	E4	13.15	< 1	2.1	0.20	9

For all the modalities, volatile acidity was higher for the non‐sulfited wines than for the sulfited wines: +0.16–0.18 g acetic acid l^−1^ for Organic modality, +0.35–0.36 g acetic acid l^−1^ for the Conventional modality and +0.12–0.16 g l^−1^ for the Ecophyto modality. The highest values were obtained for the sluggish fermentations, in which the persistence of some non‐*Saccharomyces* species is higher.

**Figure 7:**
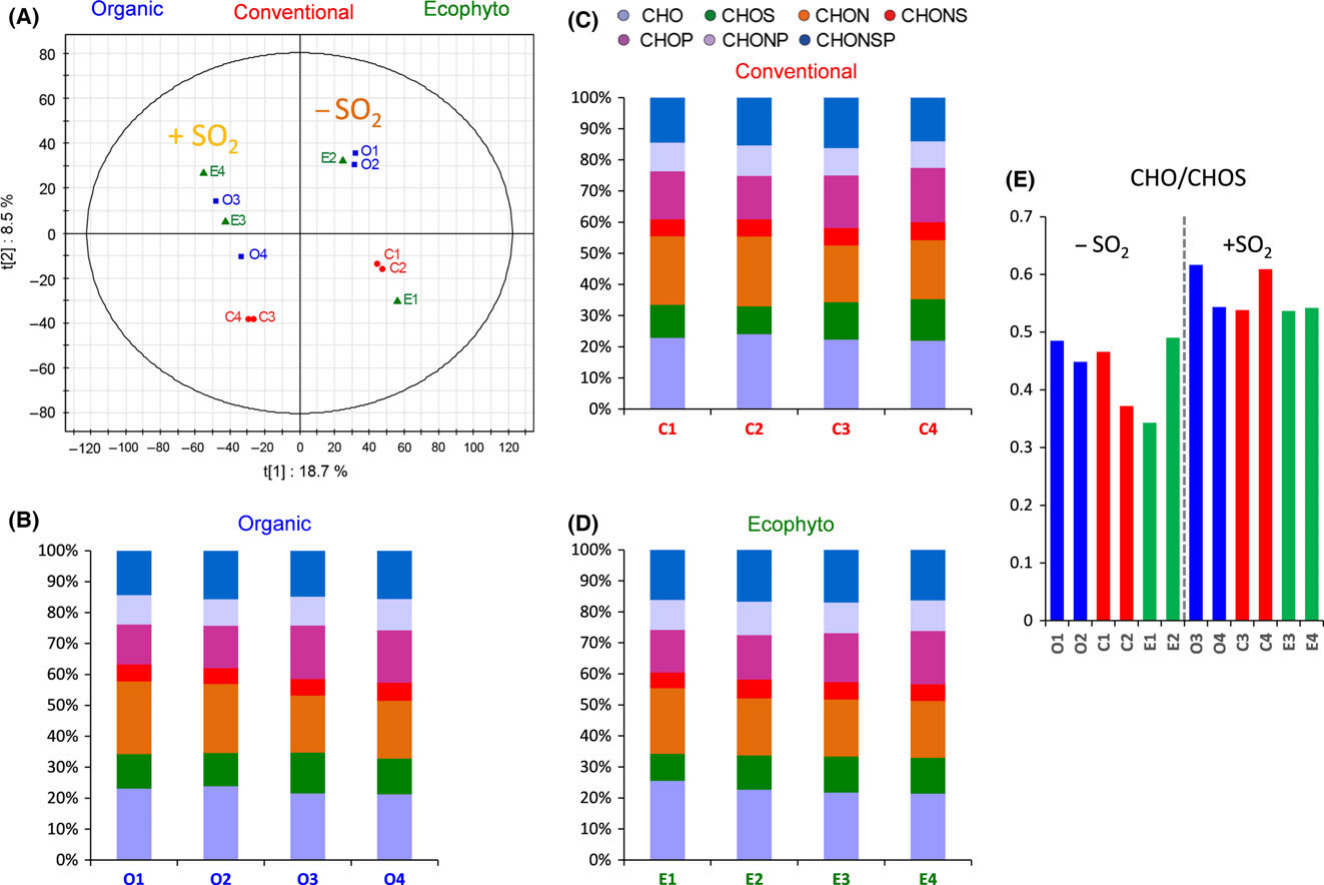
Analysis of the FT‐ICR‐MS data for wines of 2013 vintage. (A) Scores plot of the PLS‐DA depending on the use of SO2, the first two components retained 27.2% of the variation. Histograms of elementary composition of Organic (B), (C) Conventional and (D) Ecophyto wines. (E) Ratio of CHOS/CHO masses for each analysed wine.

The 12 wines were analysed by FT‐ICR‐MS. PLS‐DA was used to group the wines depending on the use of SO_2_ (Fig. 7A). Wines with SO_2_ could be separated from wines without SO_2_ along the first component (18.7% of variability). Use of SO_2_ indeed had a particular impact with a slight increase in sulfur‐containing compounds (CHOS) for the wines elaborated with sulfites (Fig. 7E). Although the overall elemental composition remained close between the different wines (Fig. 7B–D), our results show that the effects of adding sulfur dioxide to must were still detectable in wines at the end of vinification.

**Figure 8:**
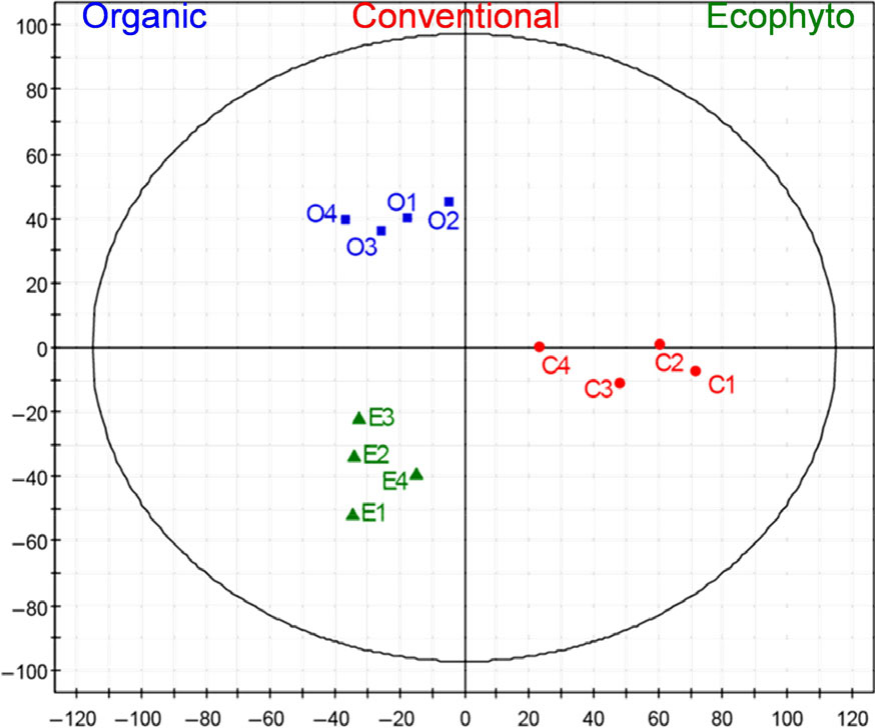
Analysis of the FT‐ICR‐MS data for wine of 2013 vintage. Scores plot of the PLS‐DA depending on phytosanitary protection mode, the first two components retained 29.4% of the variation.

However, wines can also be clearly separated by PLS‐DA (Fig. 8) according to plant protection. Thus, differences linked to plant protection were not masked by the use of SO_2_. Moreover, as already observed for the ‘terroir’ effect (Roullier‐Gall *et al*., 2014a,b), the differences related to plant protection are more visible after AF and could partly result from microbiological processes. Projecting the masses as filtered from the PLS–DA analysis on van Krevelen diagrams (Fig. S2) reveals specific chemical fingerprints for the Organic, Conventional and Ecophyto wines. It is noteworthy that almost no CHOP‐ and CHONP‐containing compounds are specific to a plant protection type. The Organic wines appear to be characterized by CHONS‐, CHONSP‐ and CHO‐containing compounds located in particular in areas of amino acids and carbohydrates according to the area of the van Krevelen diagram. The Conventional wines appear to be specifically richer in CHO‐containing compounds with some located in the carbohydrate area and by CHONS‐ and CHOS‐containing compounds. The Ecophyto wines appear to be characterized by CHONS‐, CHON‐ and CHO‐containing compounds. Thus, the existence in wines of chemical and microbiological signatures associated with plant protection is highlighted.

## Conclusion

In this study, we were able to confirm the strong influence of vintage on fungal populations on grape berries. Moreover, many interactions seemed to exist between the yeasts and mould on grape berries. Therefore, it is essential to study fungal populations of the grape as a whole to better understand the interactions involved. Furthermore, the study solely of yeasts could lead to misinterpretations: that of attributing the influence of some plant pathogenic genera to other parameters. Despite these two important factors: interactions and vintage, a significant effect of plant protection on grape populations has been highlighted with a systematic reduction of biodiversity for grapes treated in Organic modality. The use of broad spectrum fungicides based on copper in the Organic modality could be the cause of this reduction of biodiversity. However, *in vitro* studies are still required to determine the sensitivity of different fungal genera to these products as well as studies in the vineyard to determine the direct effect of the products used at the time of application.

This study also showed that fungal populations were heavily revamped during the pre‐fermentation step with a sharp reduction of mould and the presence of yeast genera not detected on berries. In spite of these reshuffles, the protection applied to the vineyard has a strong influence on fungal populations present in musts. In connection with these differences observed on grape must, populations evolve differently during AF depending on the protection applied. In addition, our results confirm the strong influence of SO_2_ on populations present during fermentation, and especially the early implantation and domination of the genus *Saccharomyces*. However, despite this selection of *Saccharomyces* yeast by the use of SO_2_, some differences in populations persisted for several days. The characterization of differences between populations at species level or intraspecies level is necessary. This could help to highlight an even greater impact on the population than that which was demonstrated in this study. Additionally, the realization of physiological testing for yeasts isolated from each must could help to better understand the mechanisms behind the influence of the protection applied.

Our results showed a significant influence of anthropogenic practices such as the use of sulfur dioxide and plant protection on the composition of wine although the compositions of the grape must from these different protections were quite similar. The wines produced could be clearly distinguished on the one hand, based on the use or not of SO_2_ and on the other hand, depending on the plant protection. It is now necessary to identify the discriminating compounds in wines elaborated for each protection to determine whether these compounds are primarily of plant or microbial origin. However, this effect is probably largely indirect and related to the modification of yeast populations during alcoholic fermentation, as the chemical differences are much more pronounced for wine than for grape must.

## Experimental procedures

### The vineyard studied

All the grapes were sampled from a plot of Chardonnay planted in 1986 and located in Burgundy, France (46°18′32.2″N, 4°44′17.9″E, 258 m altitude). Since 2007, the plot has been divided into three blocks of eight rows each: one block was managed using phytosanitary products according to conventional viticulture and noted C [pyrethroids, organophosphates, anthranilic diamides, benzamides, pyridinyl‐ethyl‐benzamides, pyridine‐carboxamides, oximino‐acetates, cyano‐imidazole, triazolo‐pyrimidylamine, triazoles, spiroketal‐amines, cinnamic acid amides, mandelic acid amides, cyanoacetamide‐oxime, phosphonates, benzophenone, dithiocarbamates, phthalimides, quinones and inorganic (sulfur and copper) fungicides]. The second block, Ecophyto (E), was managed with the same products used for the Conventional block, but with dose reduction and/or with a reduced number of treatments. Block O was managed according to organic viticulture practices for which only pyrethrins, copper and sulfur are allowed. Details on the management procedures can be found in Table S1.

### Sampling

The sampling of grape berries, bunches of grapes and total harvest were carried out in the central rows (3 and 5) (Fig. S1) of each block to overcome edge effects related to treatment. For each modality, 6 kg of ripe bunches of grapes were collected aseptically from 20 different vine plants distributed along the rows (one cluster per vine plant) for the 2012 vintage. Ten berries from each vine plant of the two rows (3 and 5) were collected for the 2013 and 2014 vintages (860 berries, 950 berries and 980 berries for the Organic, Conventionnal and Ecophyto modalities respectively). Grape berries were placed in a sterile bag and pressed manually directly in the bag. Immediately after pressing, a sample (50 ml) was taken to analyse the fungal biodiversity of the grape berries.

Moreover for the 2013 vintage, the total harvest of the two rows (3 and 5) of each modality was collected manually and placed in 20 kg crates. The harvests (102, 117 and 78 kg for modalities O, C and E respectively) were then transported to the experimental winery (Beaune, France). The harvests were pressed directly on arrival at the winery using a vertical FASER‐PLAST AG press (Rickenbach, Switzerland). The grape must thus obtained was placed overnight at 10°C for lees sedimentation (settling) and then separated into four replicates for each modality in four 20 l stainless steel vats. About 50 ml of must was sampled (T0) for each vat. The addition of sulfur dioxide (30 mg l^−1^) was carried out in two of four vats for each modality. During alcoholic fermentation, 50 ml samples were collected from each vat at various times: after 3 days (T3), 6 days (T6), 9 days (T9) and at the end of alcoholic fermentation (when density no longer decreases) (Tf). The wines obtained were then bottled without sulfiting to be used for non‐targeted chemical analysis.

### Enumeration of yeast

For each sample, serial dilutions were performed and 3 × 100 μl of each dilution were spread on the YPD medium (0.5% w/v yeast extract, 1% w/v peptone, 2% w/v glucose and 2% w/v agar supplemented with chloramphenicol at 200 ppm to inhibit the development of bacteria) and incubated at 28°C. The yeast populations were then estimated by counting the colonies developed and the result was obtained by averaging the three repetitions.

### DNA extraction

For each sample, 5 ml of must or wine was collected and centrifuged for 5 min at 4°C at 3000 ***g***. The pellet was suspended in 5 ml milliQ water and filtered through glass wool to separate cells from must debris. The filtered suspension was centrifuged again (5 min at 4°C, at 3000 ***g***). The pellet was resuspended in 200 μl of lysis buffer (2% Triton X‐100, 1% SDS, 100 mM NaCl, 10 mM Tris pH 8.1 mM EDTA pH 8), and the cells were homogenized in a bead beater (Precellys 24, France) with 0.3 g of glass beads (0.5 mm in diameter) in the presence of 200 μL phenol/chloroform/isoamyl alcohol (50:48:2). The mixture was vortexed for 1 min and placed on ice for 1 min. This step was repeated three times. Then 200 μl TE (10 mM Tris, 1 mM EDTA pH 8) was added and the bead/cell mixture was centrifuged for 10 min at 16 000 ***g*** at 4°C, after which the aqueous phase was collected. The DNA was precipitated from this aqueous phase with 2.5 volumes of 100% ethanol and centrifuged at 16 000 ***g*** at 4°C for 10 min. Then the pellet was washed with 70% ethanol, dried and suspended in 50 μl of DEPC‐treated water (Thermo Fisher Scientific, Waltham, MA, USA). The DNA concentrations of the samples were then standardized (50 ng μl^−1^) by measuring optical density at 260 nm, and adding DEPC‐treated water as appropriate before storage at −20°C.

### Pyrosequencing of 18S rRNA gene sequences

Fungal diversity was determined for each sample by using 454 pyrosequencing of ribosomal genes. A 18S rRNA gene fragment with sequence variability and appropriate size (about 350 bases) for 454 pyrosequencing was amplified using the primers FR1 (5′‐ANCCATTCAATCGGTANT‐3′) and FF390 (5′‐CGATAACGAACGAGACCT‐3′) (Chemidlin Prévost‐Bouré *et al*., 2011). For each sample, 5 ng of DNA was used for a 25 μl PCR conducted under the following conditions: 94°C for 3 min, 35 cycles of 1 min at 94°C, 52°C for 1 min and 72°C for 1 min, followed by 5 min at 72°C. A second PCR of nine cycles was then conducted under similar PCR conditions with purified PCR products and 10 base pair multiplex identifiers were added to the primers at position 5′ to specifically identify each sample and avoid PCR bias. Finally, the PCR products were purified using a MinElute gel extraction kit (Qiagen, Courtaboeuf, France) and quantified using the PicoGreen staining Kit (Molecular Probes, Paris, France). Pyrosequencing was carried out on a GS Junior apparatus (Roche 454 Sequencing System) by the GenoSol platform (INRA, Dijon, France, http://www2.dijon.inra.fr/plateforme_genosol/) and on GS FLX Titanium (Roche 454 Sequencing System) by Genoscreen (Lille, France, http://www.genoscreen.com/).

### Analysis of pyrosequencing data

Bioinformatics analyses of reads obtained by pyrosequencing were performed using the GnS‐PIPE pipeline developed by the GenoSol platform (INRA, Dijon, France) (Terrat *et al*., 2012), or the Qiime pipeline developed by scikit‐bio (Caporaso *et al*., 2010). The parameters chosen for each step were the same for the two pipelines and can be found in supplementary material (Table S2). First, all the 18S raw reads were sorted according to the multiplex identifier sequences. The raw reads were then filtered and deleted based on: (i) their length, (ii) their number of ambiguities (Ns) and (iii) their primer(s) sequence(s). A PERL program was then applied for rigorous dereplication (i.e. clustering of strictly identical sequences). The dereplicated reads were then aligned using Infernal alignment (Cole *et al*., 2009), and clustered into operational taxonomic units (OTU) using a PERL program that groups rare reads with abundant ones without counting differences in homopolymer lengths. A filtering step was then carried out to check all single‐singletons (reads detected only once and not clustered, which might be artefacts such as PCR chimeras) based on the quality of their taxonomic assignments.

The high‐quality reads retained were then taxonomically assigned using similarity approaches against dedicated reference databases from *SILVA* (Quast *et al*., 2013) (see supplementary material) (Table S2). The raw data sets are available on the EBI database system under project accession number PRJEB12990 (awaiting attribution).

Linear discriminant analysis effect size was used to determine significant taxonomic differences between the grape must sample of each phytosanitary protection (Segata *et al*., 2011). This method employs the factorial Kruskal–Wallis sum‐rank test (α = 0.05) to identify taxa with significant differential abundances between modalities (using one‐against‐all comparisons), followed by LDA to estimate the effect size of each differentially abundant feature. Significant taxa were used to generate taxonomic cladograms illustrating differences between phytosanitary protection modalities.

Shannon index (H′) was used to assess the fungal biodiversity identified by pyrosequencing in populations present on grape berries using the number of sequences to calculate *Pi*, where *i* is a genus, S the total number of genera, *n*_*i*_ the number of reads corresponding to genus *i*,* N* the total number of reads and *Pi* the proportion of genus *i* with *Pi* = *n*_*i*_/*N*:

$$H^{\prime }=\sum_{i=1}^{S}Pi\left(\log_{2}Pi\right)$$

### Oenological analysis

The dosage of reducing sugars, available nitrogenous compounds (ammonium and amino acid except proline), l‐malic acid, ethanol and acetic acid were determined by FT‐IR spectroscopy (FOSS France). pH was measured using a pH meter.

### Non‐targeted chemical analyses

Grape musts after settling and wines of the 2013 vintage were analysed by Fourier transformed ion cyclotron resonance mass spectrometry (FT‐ICR‐MS). The sample preparation consisted of a dilution of the wine in ultrapure methanol in proportions of 50 μl per 950 μl. Centrifuged grape musts were acidified by formic acid (2% v/v) to pH 2 and pre‐filtered using C18‐SPE cartridges (100 mg ml^−1^ Backerbond SPE columns) to remove sugars. C18 cartridges were conditioned by successive passages of 1 ml methanol and 1 ml of ultrapure water acidified with formic acid (1.25%). One millilitre of acidified must was then passed through the C18 cartridge by gravity, followed by 1 ml of dilute formic acid (1.25%). Finally, the acidified must was eluded with 500 μL of methanol and stored in amber vials at −20°C for analysis. Mass spectra were obtained with an FT‐ICR‐MS Solarix (Bruker Daltonics, Bremen, Germany) equipped with a 12 Tesla superconducting magnet (Magnex, UK). The instrument was equipped with an electrospray ionization source Apolo II. The samples were injected directly into a micro electrospray source at a rate of 120 μl h^−1^. The MS was externally calibrated using a 5 ppm solution of arginine (0.2 ppm tolerance). Spectra were recorded in negative ionization mode and for a mass range between *m/z* 100 and 1000. For each sample, 300 scans per sample were accumulated with a time domain of a 4 MW (megaword) per second. Spectra were then internally calibrated using a mass list of ubiquitous wine compounds with a mass error below 50 ppb. Peaks with a signal to noise ratio (S/N) of 4 and higher were used for further data processing.

Partial least square discriminative analysis (PLS–DA) models were used to provide enhanced representations of the sample category discriminations and extract the most discriminative metabolites, which were also checked manually within the spectra. Discriminative masses with a variable importance in projection (VIP) value > 2 and *P* values < 0.05 were considered as relevant. PLS‐DA was performed with the SIMCA 9 software (http://www.umetrics.com/). Two dimensional van Krevelen diagrams of discriminative metabolites were obtained using compositional networks (based on elemental compositions) and functional networks, based on selected functional group equivalents that enable improved assignment options of elemental compositions and better classification of organic complexities with tunable validation windows (Tziotis *et al*., 2011).

## Conflict of Interest

The authors declare no conflict of interest.

## Supporting information


**Fig. S1**. Satellite view (Google Earth) and plan of the experimental plot. The ranks on which the samples were taken (rows 3 and 5) are colored red.

**Fig. S2.** Analysis of the FTICR‐MS data for wine of 2013 vintage. H/C versus O/C van Krevelen diagram and related histograms of specific masses from (A) Organic wines (B) Conventional wines and (C) Ecophyto wines respectively.

**Table S1.** Doses of commercial preparations per hectare applied for each protection modes during 2012, 2013 and 2014 vintages.

**Table S2.** Bioinformatics parameters and databases used in the analysis of pyrosequencing results.

**Table S3.** Analytical characteristics of musts elaborated from grape berries harvested in three phytosanitary vineyard protections for 2013 vintage.
