# Supplementary figures and images for: Wine microbiology is driven by vineyard and winery anthropogenic factors
Source: Microb Biotechnol. 2016 Oct 25;10(2):354–70. doi: 10.1111/1751-7915.12428 (PMC5328833; doi:10.1111/1751-7915.12428)

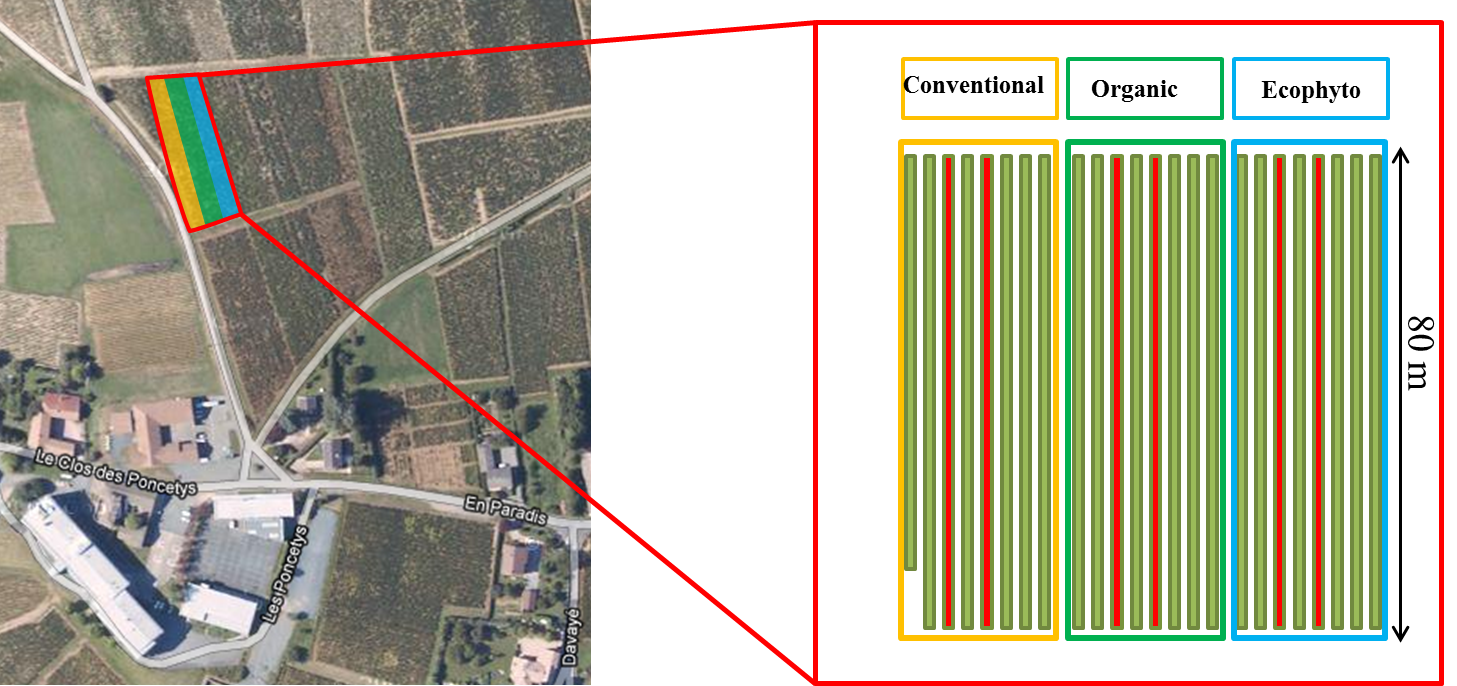

Supplement: Supplementary file 1 — Fig. S1. Satellite view (Google Earth) and plan of the experimental plot. The ranks on which the samples were taken (rows 3 and 5) are colored red. [file MBT2-10-354-s001.tif]

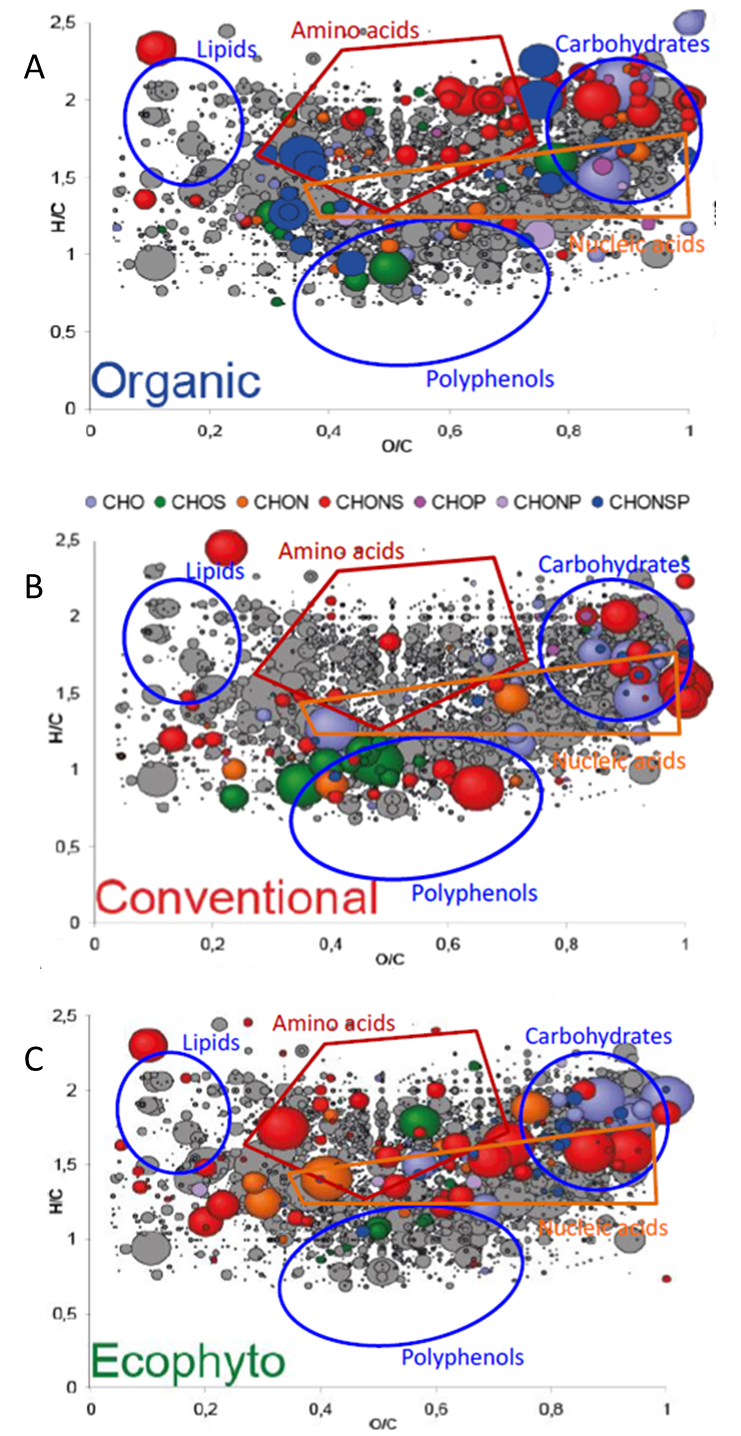

Supplement: Supplementary file 2 — Fig. S2. Analysis of the FTICR‐MS data for wine of 2013 vintage. H/C versus O/C van Krevelen diagram and related histograms of specific masses from (A) Organic wines (B) Conventional wines and (C) Ecophyto wines respectively. [file MBT2-10-354-s002.tif]
