# Supplementary material for: Wine microbiology is driven by vineyard and winery anthropogenic factors
Source: Microb Biotechnol. 2016 Oct 25;10(2):354–70. doi: 10.1111/1751-7915.12428 (PMC5328833; doi:10.1111/1751-7915.12428)
Supplement: Supplementary file 3 — Table S1. Doses of commercial preparations per hectare applied for each protection modes during 2012, 2013 and 2014 vintages. [file MBT2-10-354-s003.docx]

Table S1. Doses of commercial preparations per hectare applied for each protection modes during 2012, 2013 and 2014 vintages.

| Date | Stade of grape cultivation | O | | C | | E | |
| --- | --- | --- | --- | --- | --- | --- | --- |
|  |  | Dose applied per hectare | IFT | Dose applied per hectare | IFT | Dose applied per hectare | IFT |
| May/7/2012 | 5-7 leaves | BB RSR 1.5 kg | 0.1 | Remiltine S pépite 3 kg | 1 |  |  |
| May/16/2012 | 7-8 leaves | BB RSR 1.5 kg  Microthiol 6.25 kg | 0.6 | Sillage 4 kg  Corail 0.14 L | 1.4 |  |  |
| May/25/2012 | 9-10 leaves | BB RSR 2 kg  Microthiol 10 kg | 1 |  |  | Arco DTI 1.05 kg  Vivando 0.14 L | 1.4 |
| May/30/2012 | 10-12 leaves |  |  | Arco DTI 1.5 kg  Vivando 0.2 L | 2 |  |  |
| June/06/2012 | Flowering (beginnig | BB RSR 2.5 kg  Microthiol 10 kg | 1 |  |  |  |  |
| June/08/2012 | Flowering |  |  |  |  | Mildicut 4.5 L  Flint 0.125 kg | 1 |
| June/11/2012 | Flowering |  |  | Mildicut 4.5 L  Flint 0.125 kg | 2 |  |  |
| June/13/2012 | Flowering (end) | BB RSR 2.5 kg  Thiovit 10 kg | 1 |  |  |  |  |
| June/22/2012 | Berry peppercorn size |  |  |  |  | Valiant flash 2.1 kg | 0.7 |
| June/25/2012 | Berry 3-4 mm | BB RSR 2.5 kg  Microthiol 10 kg | 1 | Valiant Flash 3 kg  Greman 0.25 L | 2 |  |  |
| July/04/2012 | Bunch closure (beginning) | BB RSR 2 kg  Microthiol 10 kg | 1 |  |  | Sarman F 2.1 L  Prosper 0.42 L | 1.4 |
| July/09/2012 |  |  |  | Sarman F 3 L Prosper 0.6 L | 2 |  |  |
| July/16/2012 | Bunch closure (end) | BB RSR 2 kg  Microthiol 10 kg | 1 |  |  | Champflo Ampli 1.1 L Karathane3D 0.42 L | 1.3 |
| July/20/2012 | Bunch closure + 10d |  |  | Champflo Ampli 1.4 L  Karathane 3D 0.6 L | 1.7 |  |  |
| July/27/2012 | Bunch closure + 15d |  |  |  |  | Aviso cup 1.75 kg  Prosper 0.42 L | 1.4 |
| August/01/2012 | Veraison (beginning | BB RSR 2 kg  Microthiol 10 kg | 1 | Champflo Ampli 1.4 L  Prosper 0.6 L | 1.7 |  |  |
| August/10/2012 |  | BB RSR 2 kg  Microthiol 10 kg | 1 | Champflo Ampli 1.4 L  Microthiol 10 kg | 1.5 | Champflo Ampli 1.1 L | 0.6 |
| **IFT total** |  |  | **8.75** |  | **15.28** |  | **7.7** |

| Date | Stade of grape cultivation | O | | C | | E | |
| --- | --- | --- | --- | --- | --- | --- | --- |
|  |  | Dose applied per hectare | IFT | Dose applied per hectare | IFT | Dose applied per hectare | IFT |
| May/07/2013 | 3-4 leaves | Kocide Opti 0.335 kg | 0.13 | Electis Pro 1 kg | 0.5 |  |  |
| May/17/2013 | 5-6 leaves | Kocide Opti 0.67 kg Microthiol 8 kg | 0.9 | Rémiltine pépite 3 kg Prosper 0.6 kg | 2 |  |  |
| May/23/2013 | 6-7 leaves | Kocide Opti 1 kg Microthiol 8 kg | 1 |  |  |  |  |
| May/27/2013 | 7-8 leaves |  |  | Sillage 4 kg  Corail 0.4 L | 2 |  |  |
| May/31/2013 | 7-8 leaves |  |  |  |  | Prosper 0.42 L | 0.7 |
| June/03/2013 | 7-8 leaves | Kocide Opti 1 kg  Microthiol 8 kg | 1 |  |  |  |  |
| June/10/2013 | 9-10 leaves |  |  | Pergado MZ 2.5 kg  Vivando 0.2 L | 2 |  |  |
| June/13/2013 | 11-12 leaves | BB RSR 1.5 kg  Microthiol 8 kg | 1 |  |  |  |  |
| June/19/2013 | Flowering (beginning) |  |  |  |  | Corail 0.28 L  Mildicut 3.15 L | 1.4 |
| June/21/2013 |  | BB RSR 2.5 kg  Microthiol 10 kg Pyrevert 1.5 L | 2.5 |  |  |  |  |
| June/25/2013 | Flowering (end) |  |  | Enervin 2.5 kg Flint 0.125 kg  Karaté Xpress 0.25 kg | 3 | Karaté Xpress 0.25 kg | 1 |
| June/27/2013 |  | Pyrevert 1.5 L | 1 |  |  |  |  |
| July/02/2013 | Fruit set | BB RSR 2.5 kg  Microthiol 10 kg  Pyrevert 1.5 L | 2.4 |  |  |  |  |
| July/03/2013 |  |  |  |  |  | Enervin 1.75 kg Collis 0.28 L | 1.4 |
| July/08/2013 | Berry 2-3mm |  |  | Sarmant F 3 L  Corail 0.4 L Reldan 1.5 L | 3 | Reldan 1.5 L | 1 |
| July/12/2013 | Berry peppercorn size | Kocide Opti 1.33 kg  Microthiol 10 kg | 1.3 |  |  |  |  |
| July/17/2013 |  |  |  |  |  | Sarmant F 2.1 L | 0.7 |
| July/22/2013 | Bunch closure (beginning) | Kocide Opti 1.33 kg  Microthiol 10 kg | 1.3 | Sillage 4 kg  Vivando 0.2 L | 2 |  |  |
| July/31/2013 | Bunch closure | Kocide Opti 1.33 kg  Microthiol 8 kg | 1.2 |  |  | Karaté Xpress 0.5 kg  Champflo 1.1 L Corail 0.28 L | 2.3 |
| August/02/2013 | Bunch closure + 2d |  |  | Champflo 1.4 L Karathane 0.6 L  Karaté Xpress 0.25 kg | 2.7 |  |  |
| August/08/2013 | Bunch closure + 8d | Kocide Opti 1.33 kg | 0.5 |  |  |  |  |
| August/21/2013 | Veraison (beginning) | Kocide Opti 1.33 kg | 0.5 | Champflo 1.4 L | 0.7 | Champflo 1.1 L | 0.6 |
| **IFT total** |  |  | **14.91** |  | **17.9** |  | **9** |

| Date | Stade of grape cultivation | O | | C | | E | |
| --- | --- | --- | --- | --- | --- | --- | --- |
|  |  | Dose applied per hectare | IFT | Dose applied per hectare | IFT | Dose applied per hectare | IFT |
| May/06/2014 | 7-8 leaves | BB RSR 1 kg  Microthiol SPD 5 kg | 0.7 | Electis Pro 1 kg  Microthiol SPD 5 kg | 0.9 |  |  |
| May/14/2014 | 9-10 leaves |  |  |  |  | Prosper 0.42 L | 0.7 |
| May/15/2014 | 9-10 leaves | BB RSR 1.5 kg  Microthiol 8 kg | 1 | Forum top 2.5 kg  Corail 0.4 L | 2 |  |  |
| May/23/2014 |  | BB RSR 1.5 kg  Microthiol 8 kg  Pyrevert 1.55 L | 2 |  |  |  |  |
| May/27/2014 |  |  |  | Enervin 2.5 kg  Collis 0.4 L Reldan 1.5 L | 3 | Enervin 1.75 kg  Collis 0.28 L  Reldan 1.5 L | 2.4 |
| June/02/2014 |  | BB RSR 1.5 kg  Microthiol 8 kg  Pyrevert 1.55 L | 2 |  |  |  |  |
| June/11/2014 | Berry peppercorn size | BB RSR 1.5kg  Microthiol SPD 10 kg | 1.2 | Profiler 3 kg Vivando 0.2 L Magéos 0.07 kg | 3 | Magéos 0.07 kg | 1 |
| June/20/2014 |  | Microthiol SPD 10 kg | 0.8 |  |  |  |  |
| June/25/06/2014 | Fruit set (beginning) |  |  | Luna sensation 0.15 L | 1 |  |  |
| June/27/2014 |  | BB RSR 2 kg  Microthiol 8 kg  Héliosol 0.2 % | 2.2 |  |  |  |  |
| July/04/2014 | Berry 2-3 mm |  |  | Amaline Flow 2 L Coragen 0.125 L | 1.7 | Coragen 0.125 L | 1 |
| July/08/2014 | Bunch closure | BB RSR 2 kg  Microthiol 8 kg  Héliosol 0.2 % | 2.2 |  |  |  |  |
| July/18/2014 | Bunch closure + 10d | BB RSR 1,5 kg Microthiol SPD 6 kg Héliosol 0.2 % | 1.9 | Champflo Ampli 1.5 L Prosper 0.6 L | 1.8 |  |  |
| August/05/2014 | Veraison (beginning) | BB RSR 2 kg | 0.5 | BB RSR 3 kg | 0.8 | BB RSR 3 kg | 0.8 |
| **IFT total** |  |  | **14.5** |  | **14.1** |  | **5.9** |
