# Supplementary material for: Wine microbiology is driven by vineyard and winery anthropogenic factors
Source: Microb Biotechnol. 2016 Oct 25;10(2):354–70. doi: 10.1111/1751-7915.12428 (PMC5328833; doi:10.1111/1751-7915.12428)
Supplement: Supplementary file 4 — Table S2. Bioinformatics parameters and databases used in the analysis of pyrosequencing results. [file MBT2-10-354-s004.docx]

Table S2

Bioinformatics parameters and databases used in the analysis of pyrosequencing results.

| Step | Parameter Targeted rDNA gene 18S | |
| --- | --- | --- |
| Preprocessing | Length threshold | 300 |
|  | Number of ambiguities tolerated | 0 |
|  | Detection of proximal primer sequence | Complete and perfect |
|  | Detection of distal primer sequence | Perfect, but potentially incomplete |
| Clustering | Chosen level of similarity (%) | 95 |
|  | Ignoring differences in homopolymer lengths | Yes |
| Filtering | Chosen clustering similarity threshold | 95 |
|  | Used taxonomic database | SILVA (r111) |
|  | Chosen taxonomic level | Phylum |
|  | Similarity or confidence threshold (%) | 85 |
| Taxonomy | Used taxonomic database | SILVA (r111) |
|  | Method or tool of comparison | MegaBLAST |
|  | Similarity or confidence threshold (%) | 80 |
| Analysis | Chosen level of similarity (%) | 95 |
|  | Ignoring differences in homopolymer lengths | Yes |
|  | Computation of a Unifrac distance matrix | Yes |
