# Supplementary material for: Wine microbiology is driven by vineyard and winery anthropogenic factors
Source: Microb Biotechnol. 2016 Oct 25;10(2):354–70. doi: 10.1111/1751-7915.12428 (PMC5328833; doi:10.1111/1751-7915.12428)
Supplement: Supplementary file 5 — Table S3. Analytical characteristics of musts elaborated from grape berries harvested in three phytosanitary vineyard protections for 2013 vintage. [file MBT2-10-354-s005.docx]

Table S3. Analytical characteristics of musts elabored from grape berries harvested in three phytosanitary vineyard protections for 2013 vintage.

|  | Organic | Conventional | Ecophyto |
| --- | --- | --- | --- |
| glucose+fructose g/l | 209 | 212 | 211 |
| Tartaric acid g/l | 7.1 | 6.4 | 6.5 |
| pH | 3.11 | 3.10 | 3.13 |
| total acidity g tartaric acid/l | 9.2 | 8.3 | 8.3 |
| L-malic acid g/l | 5.2 | 4.9 | 5.0 |
| Available nitrogenous compounds mgN/l | 254 | 195 | 236 |
